# Evaluation of the Cytotoxic Activity of Nanostructured Lipid Carrier Systems for Fatty Acid Amides and Silk Fibroins in Breast Cancer Cell Lines

**DOI:** 10.3390/molecules30163337

**Published:** 2025-08-11

**Authors:** Sandro da Silva Borges, Sued Eustáquio Mendes Miranda, Victor Hugo de Souza Marinho, André Luís Branco de Barros, Sergio Yoshioka, Lorane Izabel da Silva Hage-Melim, Ana Carolina de Jesus Silva, Irlon Maciel Ferreira, Anna Eliza Maciel de Faria Mota Oliveira

**Affiliations:** 1 Biocatalysis and Applied Organic Synthesis Laboratory, Federal University of Amapá, University Campus Marco Zero do Equador, Rodovia Josmar Chaves Pinto, Km 02, Macapá 68902-280, AP, Brazil; ssilva.borges@hotmail.com (S.d.S.B.); virugo36@yahoo.com.br (V.H.d.S.M.); irlon.ferreira@unifap.br (I.M.F.); 2 Department of Clinical and Toxicological Analyses, Federal University of Minas Gerais, Avenida Antonio Carlos, 6627, Belo Horizonte 31270-901, MG, Brazil; sued1989@ufmg.br (S.E.M.M.); albb@ufmg.br (A.L.B.d.B.); 3 Biochemistry and Biomaterials Laboratory, Institute of Chemistry of São Carlos, University of São Paulo, Av. Trabalhador São-Carlense 400, São Carlos 13560-970, SP, Brazil; sergioy@iqsc.usp.br; 4 Laboratory of Pharmaceutical and Medicinal Chemistry, Federal University of Amapá, University Campus Marco Zero do Equador, Rodovia Josmar Chaves Pinto, Km 02, Macapá 68902-280, AP, Brazil; lorane@unifap.br (L.I.d.S.H.-M.); caroldejesus.farmacia@gmail.com (A.C.d.J.S.); 5 Laboratory of Phytopharmaceutical Nanobiotechnology, Federal University of Amapá, University Campus Marco Zero do Equador, Rodovia Josmar Chaves Pinto, Km 02, Macapá 68902-280, AP, Brazil

**Keywords:** *Bombyx mori*, breast cancer, andiroba, cannabinoid receptor, Biomaterials, cytotoxicity

## Abstract

Breast cancer, a highly prevalent malignancy among women, continues to pose a significant global health challenge, as conventional therapies are often limited by adverse effects. This study developed and evaluated nanostructured lipid carriers (NLCs) encapsulating fatty acid amides (FAAs) semi-synthesized from andiroba oil and combined with silk fibroin (SF) as a novel therapeutic strategy. Methods: FAAs were synthesized via direct amidation and characterized by GC-MS, FT-IR, and ^13^C NMR. These fatty acid amides were then incorporated into NLCs containing SF. The formulation was evaluated for its physicochemical stability, cell selectivity, and cytotoxicity against 4T1 murine breast cancer cells and healthy human fibroblasts. Results: The NLC-FAA/SF formulation exhibited physicochemical stability (average particle size: 136.9 ± 23.6 nm; zeta potential: −8.3 ± 12.0 mV; polydispersity index: 0.19 ± 0.04), indicating a monodisperse and stable system. In vitro cytotoxicity assays demonstrated high selective activity against 4T1 murine breast cancer cells (IC_50_ = 0.18 ± 0.06 μg/mL) and negligible toxicity to healthy human fibroblasts. Molecular docking studies revealed favorable interactions between the FAAs and cannabinoid receptors CB1 and CB2, with unsaturated FAAs showing higher binding scores and stability, suggesting their potential as cannabinoid receptor ligands. These findings highlight NLC-FAA/SF as a promising, selective, and effective nanoplatform for breast cancer treatment, warranting further investigation into its mechanism of action and in vivo efficacy.

## 1. Introduction

Cancer is a complex disease characterized by uncontrolled cell growth and metastasis, representing a significant global health burden [1]. It is not a single disease but rather a term encompassing over 100 different types of cancer, classified based on the cell type where the disease originates. Among these, breast cancer remains a leading cause of mortality among women, with millions of new cases diagnosed annually [2].

In 2021, the age-adjusted breast cancer mortality rate for the global population was 11.71 deaths per 100,000 women [3]. According to this estimate, the incidence of breast cancer by the year 2050 is expected to increase by up to 32.13%, reaching 59.63 cases per 100,000 women, thereby becoming the leading cause of cancer-related death among women [4].

Generally, metastatic tumors are treated with chemotherapy drugs that inhibit cell proliferation [5]. However, most of these drugs are non-specific and can cause numerous side effects by affecting both malignant and normal cells. This necessitates the exploration of novel, more selective, and less toxic therapeutic approaches, particularly those leveraging natural compounds and advanced drug delivery systems [6].

Fatty acid amides (FAAs), both natural and synthetic, have garnered attention for their potential anticancer properties [7]. They are easily synthesized through direct amidation [8] or by modifying long-chain fatty acid esters or acids [9,10]. FAAs also exhibit lower cellular toxicity compared to other bioactive compounds [11]. Studies on anandamide [12], palmitoylethanolamide [13], N-(2-hydroxyethyl) hexadecanamide [14], and other FAAs like fatty acid benzylamides [15] highlight their pharmacological potential in treating breast cancer (BC).

FAAs are capable of suppressing tumors via the P53 regulatory gene, proapoptotic gene, and antiapoptotic gene (downregulation), in addition to inducing apoptosis through the upregulation of the cell cycle regulator P21 via cannabinoid receptors in malignant neoplasms [14]. This interaction with cannabinoid receptors is important because high CB1R expression within the endocannabinoid system (eCB) may be associated with a poor prognosis in the HER2+ breast cancer subtype [16].

Recent insights into cancer pathophysiology have highlighted the endocannabinoid system (eCB) and its receptors, CB1 and CB2, as potential therapeutic targets. Although not direct oncogenic drivers, these receptors are expressed in breast cancer cells and are involved in key processes such as cell proliferation, migration, invasion, and apoptosis, making them relevant subjects for further investigation. Fatty acid amides (FAAs), due to their affinity for the eCB system, are considered cannabinomimetic or cannabinoid-like compounds, capable of interacting with both CB1 and CB2 receptors [17]. Both receptors, members of the G protein-coupled receptor family (Gi/o) [18,19], attenuate cellular responses by inhibiting adenylate cyclase activity [20,21].

Due to their lipophilic nature, FAAs have limitations in aqueous environments. To overcome these challenges and enhance the therapeutic index of FAAs, nanostructured lipid carriers (NLCs) have emerged as a highly promising drug delivery platform. These systems are capable of encapsulating and releasing varying concentrations of FAAs into target cells, as they offer superior encapsulation efficiency, controlled release profiles, and improved stability for lipophilic compounds without altering the physicochemical properties of the biocompounds [22,23]. In this context, studies with the prodrug FAA nucleoside analog gemcitabine incorporated into nanoparticles demonstrated high drug concentration and increased surface-to-mass ratio, resulting in enhanced release and improved antitumor effects [24].

Furthermore, the incorporation of natural biopolymers like silk fibroin (SF), a biocompatible and biodegradable protein from *Bombyx mori*, can enhance the stability and bioavailability of nanostructured lipid carriers (NLCs). SF has demonstrated low immunogenicity and favorable structural properties, making it a promising material for drug delivery systems [25,26].

In this context, the present study evaluated the selectivity and antitumor activity of NLCs, prepared using silk fibroin and fatty acid amides semi-synthesized from andiroba oil (AO) as a source of fatty acid chains, in the 4T1 murine breast cancer cell line and human fibroblasts.

## 2. Results

### 2.1. Evaluation of Semi-Synthesis and Spectroscopic Characterizations

After reaction and purification, the triglycerides present in andiroba oil were converted into the respective FAAs. The primary FAAs identified were *N*-isobutylpalmitamide (40%) and *N*-isobutylstearamide (4%), both saturated chain amides, as well as *N*-isobutyloleamide (44%) and (9Z,12Z)-N-isobutyloctadeca-9,12-dienamide (2%), which are derived from linoleic acid and represent unsaturated chain amides. Additionally, 10% of unidentifiable compounds were detected. The relative percentage of FAA was calculated based on the corresponding peak integrator areas and identified using a mass spectrometry (MS) database (NIST 5.0) and the literature [27].

**Figure 1:**
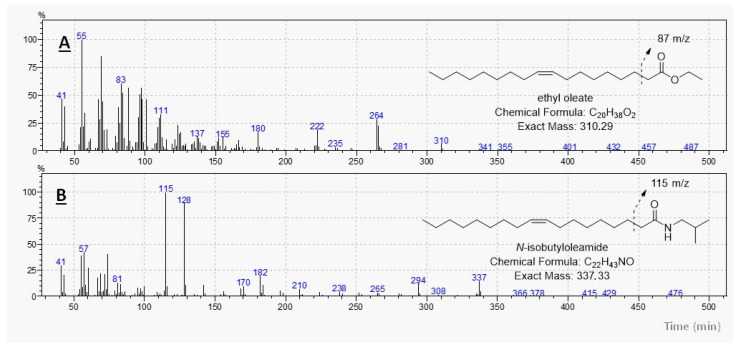
Mass spectrum (70 eV) of the (**A**) Ethyl oleate and (**B**) N-isobutyloleamide.

The fragmentation profile of the fatty isobutylamides was determined using electron impact mass spectrometry (MS) (70 eV), with the base peak observed at a mass-to-charge ratio of 115 m/z, resulting from the McLafferty rearrangement. As shown in the fragmentation spectrum of N-isobutyloleamide, the molecular ion was detected at 337 m/z, with a base peak at 115 m/z (Figure 1).

**Figure 2:**
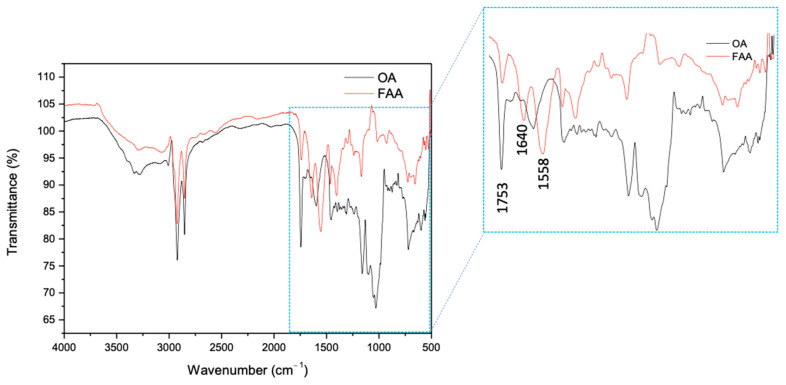
Infrared spectrum of OA (black) and FAA (red).

The corresponding infrared spectra are shown in Figure 2. Due to the presence of saturated and unsaturated alkyl chains derived from the AO triglyceride, spectra A and B exhibit a high degree of similarity. The peak at 3005 cm^−1^ corresponds to the stretching of the –C–H (sp^2^) bond, while the peaks at 2924 and 2854 cm^−1^ correspond to the stretching of the –C–H (sp^3^). Another characteristic peak of triglyceride-derived compounds appears at 1753 cm^−1^, attributed to the carbonyl C=O bond stretching of the AO and FAA. The signals for the amidated compound are observed as peaks at 3385 and 3304 cm^−1^, which correspond to the stretching of the –NH_2_ group, and the peaks at 1640 and 1558 cm^−1^ are associated with the bending of the–N–H bond, characteristic of the synthesized fatty amide. Conventionally, the peak at 3385 cm^−1^ was attributed to the free NH group in amides, and the peak at 1558 cm^−1^ was linked to NH_2_, corresponding to the symmetrical in-plane bending deformation, confirming the identity of the compound [28].

**Figure 3:**
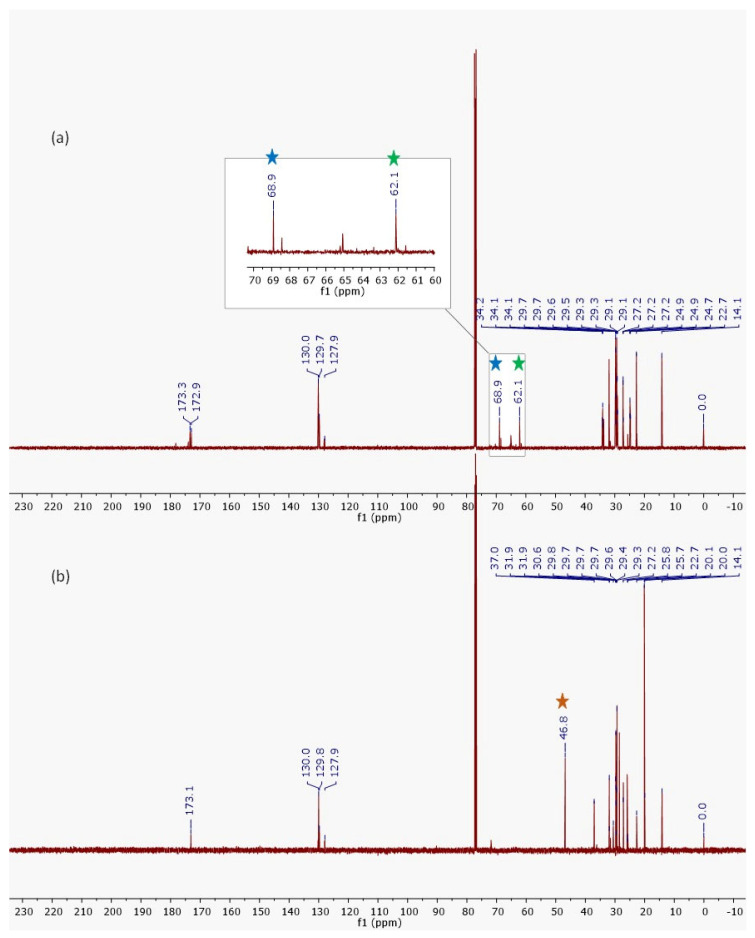
NMR spectrum of ^13^C from AO (**a**) and FAAs (**b**).

The AO and the FAAs exhibited very similar ^13^C NMR signals (Figure 3), attributed to the retention of the alkyl chain from the initial material (AO) and its preservation in the resulting products. The primary distinction in the ^13^C NMR spectrum occurs at 62.1 and 68.9 ppm, which are characteristic of the glycerol carbons present in AO (star blue). However, the ^13^C NMR spectrum of the FAA mixture displayed a prominent signal at 46.8 ppm (Figure 3b/star yellow), corresponding to the carbon of the -N–CH2 bond (Figure 3b/star yellow), and the absence of glycerol signals further indicated the formation of a new compound derived from AO.

### 2.2. Characterization and Evaluation of the Physicochemical Stability of NLCw and NLC-FAA/SF

**Figure 4:**
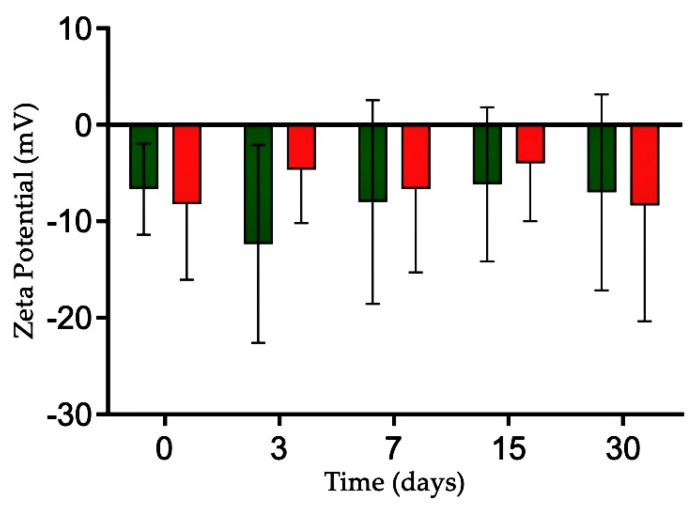
Graphical representation of the physicochemical stability concerning the size, polydispersion, and zeta potential of the NLCw (green) and NLC-FAA/SF (red) for the times 0, 3, 7, 15, and 30 days.

Polymeric nanoformulations are critical components of nanometer-sized drug carriers [29]. The NLC-FAA/SF nanocarrier and the NLCw were evaluated on days 0, 3, 7, 15, and 30 after preparation in terms of particle size, polydispersity index (PdI), and zeta potential (ZP) using dynamic light scattering (DLS), which is one of the best methods to assess the temporal stability of a nanosystem. Throughout the evaluation period, no significant changes were observed (Figure 4).

The NLC-FAA/SF exhibited particle sizes ranging from 130.90 ± 8.61 to 136.9 ± 23.6 nm (Table 1), while the NLCw ranged from 176.70 ± 18.07 nm to 157.2 ± 14.4 nm. The NLC-FAA/SF showed PdI values between 0.21 ± 0.04 and 0.19 ± 0.04, while NLCw showed PoI values between 0.20 ± 0.04 and 0.20 ± 0.04.

The zeta potential of NLC-FAA/SF ranged from −8.33 ± 7.64 mV to −8.3 ± 12.0 mV, while NLCw ranged from −6.67 ± 4.73 mV to −7.0 ± 10.1 mV over the 30-day storage period.

The results of the physicochemical evaluation of the proposed NLC formulations, with and without FAA/SF, showed that both formulations exhibited low polydispersity index values (≤0.3) and small average particle sizes, indicating a monodisperse distribution [30].

**Figure 5:**
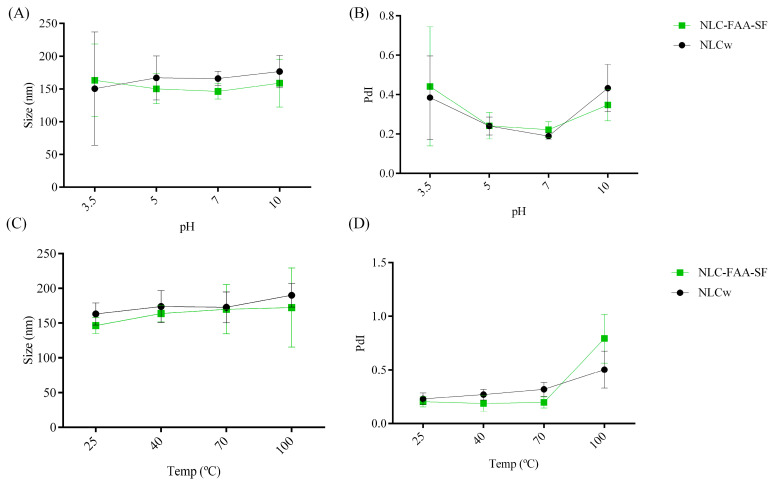
Evaluation of the influence of pH on particle size (**A**) and polydispersity index (PdI) (**B**), and the influence of temperature on particle size (**C**) and PdI (**D**).

A trend toward changes in droplet size (Figure 5) was observed as the temperature increased, although no statistically significant alterations were detected. This behavior may be associated with the possible migration of some substances from the droplets to the external phase, as temperature positively affects solubilization, followed by an additional loss of more volatile substances. Regarding the polydispersity index, a tendency for a decrease was observed as the temperature increased in the concentrated systems, although this change was considered not significant (0.169 ± 0.014 to 0.206 ± 0.01). In this context, particle sizes exceeding 200 nm can significantly affect circulation time, as this is influenced by the capillary size of the circulatory system.

The ZP evaluates the electrostatic repulsion between equally charged particles. It is important to clarify that while ZP values above +30 mV or below −30 mV are generally considered indicative of high colloidal stability due to strong electrostatic repulsion, nanoparticle stability is not governed solely by ZP. In our formulation, the NLC-FAA/SF system exhibited ZP values ranging from −8.33 ± 7.64 mV to −8.3 ± 12.0 mV, which may appear low under the criteria for electrostatic stabilization. However, these values must be interpreted considering the nature of the stabilizing agents used. The formulation includes Tween 80, a non-ionic surfactant, and silk fibroin, an amphiphilic protein that forms a viscoelastic steric barrier at the oil–water interface. This steric stabilization mechanism is effective even at lower ZP values, as it prevents particle aggregation through spatial hindrance rather than electrostatic repulsion. The stability of the formulation is further supported by the absence of significant changes in particle size and polydispersity index over 30 days of storage (Figure 4). Therefore, the observed physical stability results from a combined steric and mild electrostatic stabilization, as supported by previous literature, and should not be assessed based solely on ZP thresholds [31]. Additionally, long organic chains, such as those of the FAA present in the formulation, can create a steric barrier that prevents interaction between the nanoparticles [32].

A trend toward changes in droplet size (Figure 5) was observed as the temperature increased, although no statistically significant alterations were detected. This behavior may be associated with the possible migration of some substances from the droplets to the external phase, as temperature positively affects solubilization, followed by an additional loss of more volatile substances. Regarding the polydispersity index (PdI), a tendency for a decrease was observed as the temperature increased in the concentrated systems, although this change was considered not significant (0.169 ± 0.014 to 0.206 ± 0.01).

**Figure 6:**
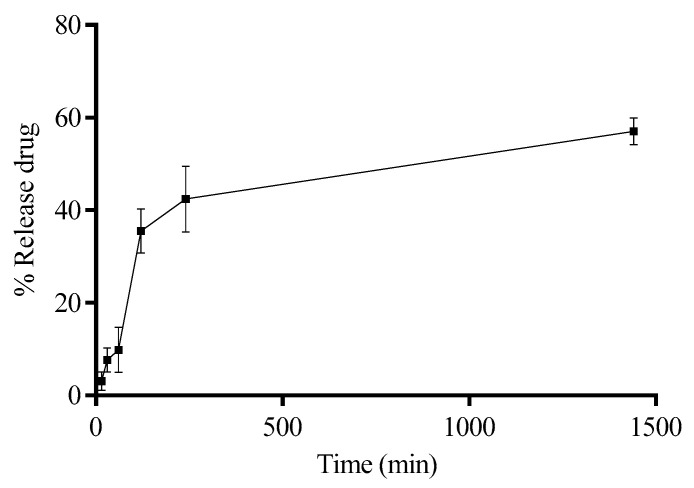
Drug release profile of FAA from NLC at 37 °C for 24 h.

Quantification using infrared spectroscopy has been increasingly utilized in the pharmaceutical field. A method using ATR-FTIR spectroscopy was employed for measuring the release of the FAA from a nanoemulsion. The measurements focused on the areas under IR bands at 2955, 2922, 1653, 1545, and 1239 cm^−1^ [33]. The calibration showed good linearity with correlation coefficients (R) above 0.995, and the determination coefficient (R^2^) was above 0.991. Precision was supported by intra-day RSDs up to 3% and inter-day RSDs up to 8%. Recovery tests gave values between 92% and 108%, confirming the method’s accuracy. No interfering peaks appeared in the blank or placebo samples, which showed specificity. The lowest amounts detected and quantified were 0.06 µg/mL and 1.02 µg/mL, respectively. Stability tests showed changes in peak areas staying within ±8% over time. The ratio of the 2922 cm^−1^ to 2955 cm^−1^ bands was also calculated to see how fatty acid chains changed during release. Overall, the method proved reliable for studying the release of fatty acid amides from the nanoemulsion [34,35,36]. In this study, we employed this method to quantify fatty acid amides encapsulated in silk fibroin nanoparticles. Our results demonstrated that the system effectively modulated the release profile, maintaining approximately 40% release after 4 h of study (Figure 6). Moreover, even after 24 h, the system released only 57% of the amides encapsulated in the core, indicating a sustained release behavior.

### 2.3. Evaluation of Colloidal Stability in Biological Media

In our study, the nanosystem’s interactions with various media were assessed to simulate administration conditions. A slight, non-significant increase in particle size was observed at the final evaluation, remaining within US Pharmacopeia limits [37,38].

**Figure 7:**
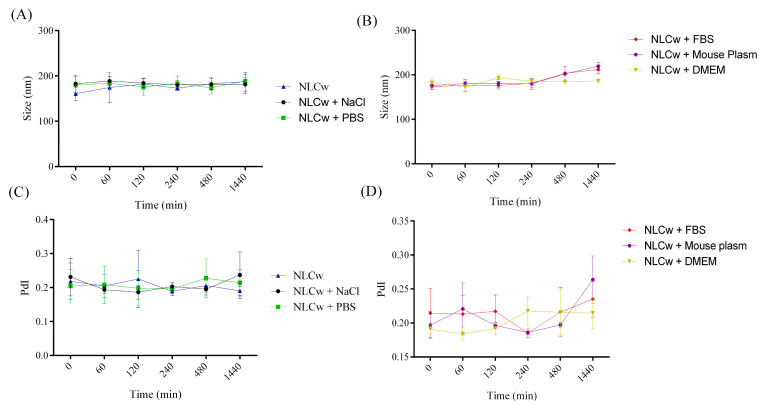
Size (**A**,**B**) distribution and polydispersion (**C**,**D**) of NLCw in the absence of FAA and SF for times 0, 60, 120, 240, 480, and 1440 min, in different biological media.

**Figure 8:**
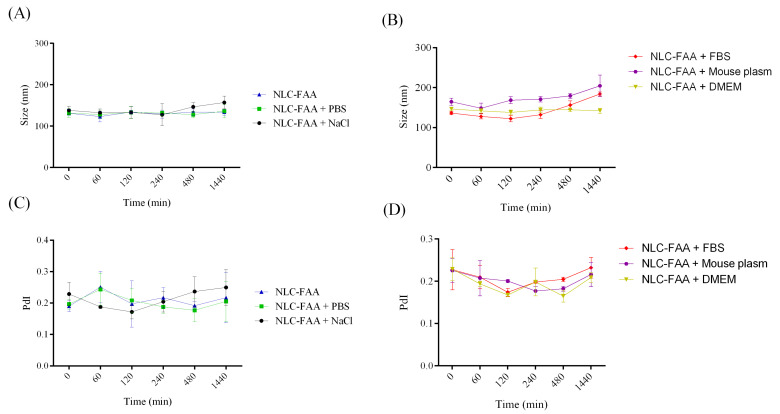
Size (**A**,**B**) distribution and polydispersion (**C**,**D**) of NLC-FAA/SF for times 0, 60, 120, 240, 480, and 1440 min, in different biological media.

The NLCs-FAA/SF system demonstrated robust physicochemical stability, reduced nanoparticle aggregation, and effective encapsulation of lipophilic agents such as fatty acid amides (FAAs). Colloidal stability is a crucial parameter to evaluate when considering potential in vivo administration, as it is essential for identifying new pharmacological alternatives. Therefore, NLCw and NLC-FAA/SF were exposed to the following media: phosphate buffer (PBS), 0.9% NaCl saline, fetal bovine serum (FBS), mouse plasma, and culture medium (DMEM), over 24 h (Figure 7 and Figure 8).

There were no significant differences in the size distribution of NLCw over time, regardless of the medium used to simulate in vivo administration. The NLC-FAA/SF exhibited a different behavior, with a slight increase in size distribution after two hours of incubation in plasma. Additionally, it is worth noting that the FBS medium showed a similar profile.

The results concerning particle size and PdI over the 24 h for NLCw and NLC-FAA/SF are shown in Table 1.

### 2.4. In Vitro Cytotoxicity

**Figure 9:**
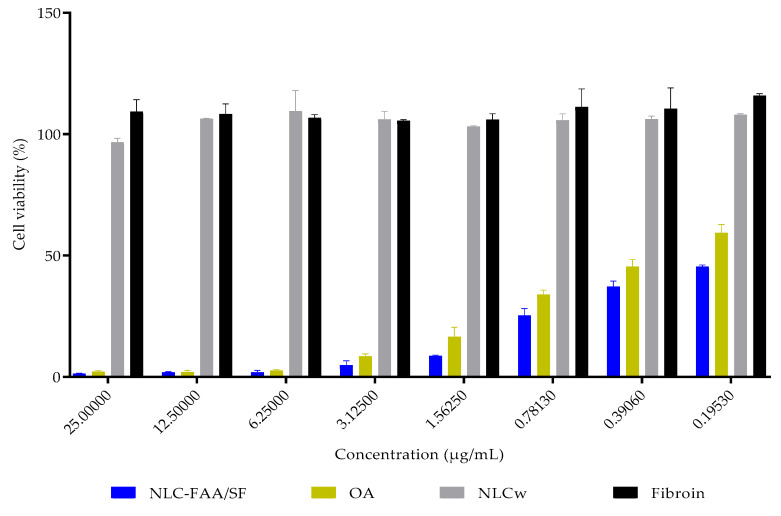
Viability in 4T1 murine breast cancer cells. NLC-FAA/SF—Nanostructured Lipid Carrier containing fatty acid amide and silk fibroin; OA—Andiroba Oil; NLCw—White Nanostructured Lipid Carrier.

In vitro, NLC-FAA/SF demonstrated potential as a formulation for treating breast tumors, as the IC_50_ viability in 4T1 murine breast cancer cells was achieved with a concentration 56.25% lower than that of free andiroba oil, as shown in Figure 9 and Table 2.

**Figure 10:**
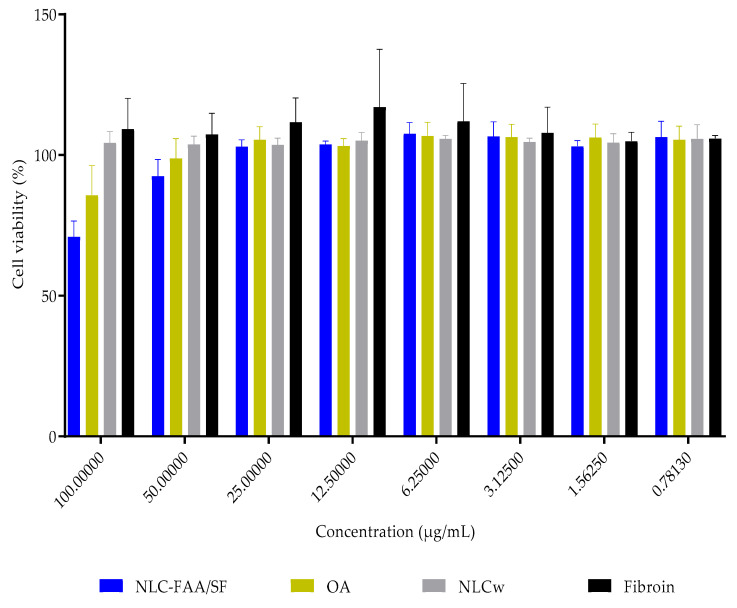
Viability in healthy human fibroblasts. NLC-FAA/SF—Nanostructured Lipid Carrier containing fatty acid amide and silk fibroin; OA—Andiroba Oil; NLCw—White Nanostructured Lipid Carrier.

The IC_50_ value could not be determined for healthy fibroblasts even at the highest tested concentrations (137.8 ± 9.9 μg/mL) and in AO (147.7 ± 7.9 μg/mL). Similarly, none of the concentrations of NLCw tested without FAA/SF or fibroin reduced the viability of healthy fibroblasts (Figure 10 and Table 3).

### 2.5. Molecular Docking

The study of different molecules with varied structures is essential for a better understanding of the potential effects that may arise when interacting with these targets and what possible effects may be triggered by such interactions. In this context, molecular docking studies were conducted to investigate the interactions between fatty acid amides (FAAs) and cannabinoid receptors CB1 and CB2, which are relevant targets in the context of breast cancer due to their role in modulating tumor-related processes and interacting with the endocannabinoid system.

Docking method validation demonstrated reproducibility and accuracy, with RMSD values of 0.823 Å for CB1 and 1.46 Å for CB2, both within the acceptable threshold of 2 Å.

**Figure 11:**
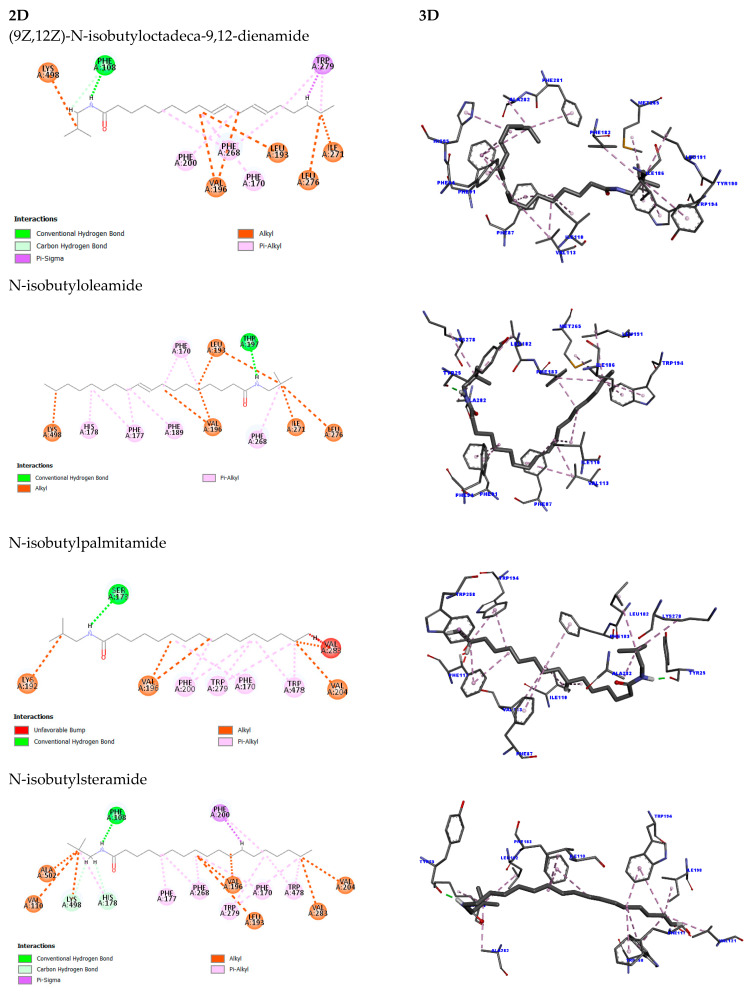
The 2D and 3D representation of the molecular docking simulation for the best pose of each fatty acid amide at the CB1 target.

Molecular docking between the molecule (9Z,12Z)-N-isobutyloctadeca-9,12-dienamide and the CB1 target revealed a total of seventeen interactions. These included two hydrogen bonds involving the Phe108 residue—one conventional and one carbon–hydrogen bond (Figure 11)—which require proximity and result in stronger interactions due to the small size and polarity of hydrogen atoms [39]. The remaining interactions were hydrophobic: one pi-sigma interaction with Trp279 (between its aromatic ring and H49 of the ligand), six alkyl interactions with Val196, Leu193, Ile271, Leu276, and Lys498, and eight pi-alkyl interactions with Phe170, Phe200, Phe268, and Trp279. The docking score, reflecting binding affinity, was 95.19.

Regarding the docking between CB1 and *N*-isobutyloleamide, the score was 95.71, and it exhibited a total of sixteen interactions, with only one conventional hydrogen bond with the Thr197 residue. The other interactions were all hydrophobic, with seven alkyl-type interactions involving the residues Val196, Leu193, Lys498, Ile271, and Leu276 and seven pi-alkyl-type interactions with the residues Phe170, Phe177, His178, Phe189, and Phe268.

In contrast, N-isobutylpalmitamide exhibited lower affinity (score: 71.94), possibly influenced due to four unfavorable interactions with Val283. These were caused by the high proximity between the ligand and the target at the site, and due to the size of the atoms and their spatial arrangement, these interactions have a low probability, as seen in Table 1, where the distance between the molecules is shorter. The other interactions for the *N*-isobutylpalmitamide amide included one hydrogen bond between Ser173 and H54 of the ligand, of the conventional type, and twelve hydrophobic interactions, five of the alkyl type with the residues Val196, Val204, Val283, and Lys192 and six of the pi-alkyl type, with the residues Phe200, Trp279, and Trp478.

For N-isobutylsteramide, the affinity with CB1 was similar (score: 71.71), despite twenty-three interactions. Of these, three were hydrogen bonds: one of the conventional type with the Phe108 residue and two of the carbon–hydrogen type with the amino acids His178 and Lys498. Regarding the hydrophobic interactions, one pi-sigma interaction was observed with the Phe200 residue and eight alkyl interactions involving the amino acids Val196, Ala502, Leu193, Val204, Val283 and Lys498. Finally, eleven pi-alkyl interactions were observed with the residues Phe170, Phe177, His178, Phe200, Phe268, and Trp279.

Molecular docking studies were conducted to evaluate the interactions between fatty acid amides (FAAs) and cannabinoid receptors CB1 and CB2, both of which are relevant in breast cancer due to their role in tumor progression and the endocannabinoid system. The docking protocol was validated by re-docking the original ligands, yielding RMSD values of 0.823 Å for CB1 and 1.46 Å for CB2, confirming accuracy and reproducibility (raw data are displayed in Supplementary Materials, Table S1).

The FAA (9Z,12Z)-N-isobutyloctadeca-9,12-dienamide showed a docking score of 95.19 with CB1, forming seventeen interactions, including two hydrogen bonds with Phe108, one pi-sigma interaction with Trp279, six alkyl interactions, and eight π-alkyl interactions. N-isobutyloleamide demonstrated a slightly higher docking score (95.71) with sixteen interactions, including one hydrogen bond (Thr197) and a variety of hydrophobic interactions with Val196, Leu193, Lys498, and aromatic residues such as Phe170 and Phe268 (Figure 11).

Saturated amides exhibited lower binding affinity. N-isobutylpalmitamide showed a docking score of 71.94 and formed one hydrogen bond (Ser173), with twelve hydrophobic interactions and four unfavorable contacts with Val283. N-isobutylstearamide displayed a similar score (71.71), forming three hydrogen bonds (including one with Phe108) and twenty hydrophobic interactions, with several contacts involving Phe170, Phe177, and Trp279.

**Figure 12:**
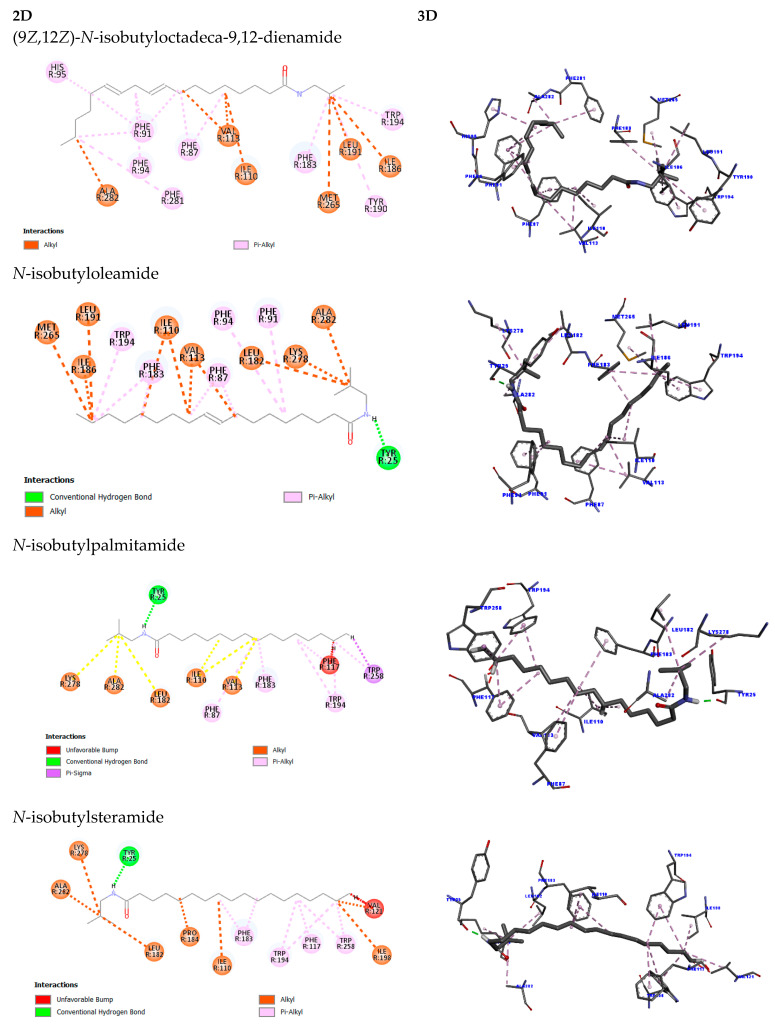
The 2D and 3D representation of the molecular docking simulation of the best pose of each fatty acid amide on the CB2 target.

Docking results for CB2 mirrored the trend observed for CB1. (9Z,12Z)-N-isobutyloctadeca-9,12-dienamide showed the highest affinity (score: 91.02), forming twenty hydrophobic interactions (seven alkyl, thirteen π-alkyl). N-isobutyloleamide followed closely with a score of 88.58, exhibiting one hydrogen bond (Tyr25) and eighteen hydrophobic interactions. In contrast, N-isobutylpalmitamide and N-isobutylstearamide demonstrated significantly lower scores (53.34 and 51.28, respectively), with fewer favorable and more unfavorable interactions (Figure 12).

Just as in the CB1 receptor, the same pattern was observed for the CB2 receptor, where the first two fatty acid amides (9*Z*,12*Z*)-*N*-isobutyloctadeca-9,12-dienamide and *N*-isobutyloleamide showed superior results in terms of affinity when compared to the last two (raw data are displayed in Supplementary Materials, Table S2).

The molecular docking analysis of fatty acid amides with the CB2 receptor revealed variable binding affinities. (9Z,12Z)-N-isobutyloctadeca-9,12-dienamide exhibited the highest binding score (91.02), forming twenty hydrophobic interactions, including seven alkyl-type interactions (Val113, Ala282, Ile110, Ile186, Leu191, Met265) and thirteen π-alkyl interactions (Phe87, Phe91, Phe94, His95, Phe183, Tyr190, Trp194, Phe281).

N-isobutyloleamide presented a slightly lower score (88.58), forming nineteen interactions, including one conventional hydrogen bond (Tyr25) and eighteen hydrophobic interactions—ten of the alkyl type (Val113, Ala282, Ile110, Ile186, Leu191, Met265, Leu265, Lys278) and eight of the π-alkyl type (Phe87, Phe91, Phe94, Phe183, Trp194).

In contrast, the saturated amides exhibited significantly lower affinities. N-isobutylpalmitamide obtained a docking score of 53.34, forming one hydrogen bond (Tyr25) and thirteen hydrophobic interactions, including one π–σ interaction (Trp258), six alkyl interactions (Val113, Ala282, Ile110, Leu182, Lys278), and seven π-alkyl interactions (Phe87, Phe117, Phe183, Trp194, Trp258). Additionally, unfavorable interactions were observed with Phe117.

Finally, N-isobutylstearamide showed the lowest binding score (51.28), with one hydrogen bond (Tyr25) and fourteen hydrophobic interactions (seven alkyl: Pro184, Ala282, Ile110, Val121, Ile198, Leu182, Lys278; seven π-alkyl: Phe117, Phe183, Trp194, Trp258), as well as four unfavorable interactions with Val121. These findings suggest that the presence of unsaturation in FAAs enhances both the affinity and stability of interactions with the CB2 receptor.

According to Hua et al. (2017), the residues Phe200 and Trp356 are critical for CB1 receptor activation through a conformational mechanism termed the “twin toggle switch” [40]. Additionally, interactions within the hydrophobic core—specifically involving Phe268, Phe397, Phe189, Phe177, Ser383, Leu193, Val196, Tyr275, Leu276, Leu359, and Met363—may contribute significantly to receptor modulation. In the present molecular docking study, several of these residues were engaged by fatty acid amides (FAAs), suggesting potential involvement in receptor activation.

Previous work by Pawar et al. (2022) demonstrated curcumin’s agonistic potential at CB2 through docking and in vivo assays, identifying interactions with 17 residues including Phe87, Ser90, Phe91, Lys109, Ile110, Val113, Thr114, Phe117, Phe183, Ile186, Tyr190, Leu191, Trp194, Trp258, Met265, Ser285, and Cys288 [41]. Similarly, Omar et al. (2022) highlighted residues such as Phe183, Thr114, Leu182, and Ser285 as relevant for CB2 activation [42].

Aviz-Amador et al. (2021), through virtual screening and molecular dynamics, identified the analog LS-61176 as a potential CB1 negative allosteric modulator and CB2 agonist, analogous to THC and CBD [43]. For CB1, key interacting residues included Met103, Asp104, Ile105, Phe108, Leu196, Val196, Phe268, Met384, and Ala380. CB2 interactions involved Phe87, Phe91, Phe94, His95, Phe106, Phe110, Phe183, and Phe281. For cannabidiol, CB1 interactions encompassed Phe102, Met103, Phe170, Val196, Phe263, Ile271, Tyr275, Leu276, Trp279, Phe379, Ser383, Cys286, and Leu387; CB2 interactions involved Ile27, Phe87, Phe94, His95, Phe106, Lys109, Ile110, Val113, Phe183, Pro184, and Lys278.

The FAAs assessed in this study showed interaction profiles consistent with these findings, engaging key residues previously reported as functionally relevant in CB1 and CB2 signaling. This supports the structural validity of the docking targets and suggests potential bioactivity of the FAAs through canonical binding mechanisms. Notably, unsaturated FAAs exhibited higher binding scores and an absence of unfavorable interactions compared to saturated analogs. This pattern indicates that molecular unsaturation may enhance binding stability by conferring conformational rigidity, thus improving fit within the receptor’s active site.

Despite their pharmacological promise, the clinical application of FAAs is limited by poor aqueous solubility. Encapsulation within lipid-based nanosystems offers a viable strategy to enhance bioavailability, prolong circulation time, and enable tumor-targeted delivery [44,45]. Such platforms also facilitate co-encapsulation of synergistic agents, potentially amplifying antitumor efficacy or minimizing adverse effects [46].

In summary, FAAs—particularly those with unsaturated structures—demonstrate favorable binding to key CB1 and CB2 receptor residues. Combined with nanocarrier-based delivery strategies, these compounds represent a promising avenue for the development of targeted therapies in breast cancer and other malignancies.

## 3. Discussion

The direct amidation reaction follows the principles of a transesterification reaction, occurring in three consecutive steps and yielding fatty amides and glycerol as a by-product [8]. The success of the reaction is dependent on several factors, including reaction conditions (temperature, catalyst, molar ratio, reaction time, and the nucleophilic species) [47]. Isobutylamine, as the primary amine, exhibits favorable reactivity in the direct transamidation process. Consequently, it has been observed that the proportion of the fatty chain (saturated/unsaturated) in the respective andiroba oil remains unchanged following the transamidation reaction. To assess the toxicity potential of fatty amides in 4T1 cells, we sought to better understand the influence of the fatty chain (unsaturated/saturated) derived from the direct amidation process, with the alkyl chain sourced from the fatty acids present in andiroba oil. It is important to note that this semi-synthetic method of obtaining fatty amides is considered the most feasible approach, as it can reduce production costs and make the process attractive for large-scale production [48,49]. While evaluating isolated substances is critical, this study investigates the cellular toxicity of the fatty amide mixture in 4T1 cells.

For the development of the nanostructures, the phase inversion temperature (PIT) technique was employed. The effect of this technique is linked to the interaction between the surfactant’s spontaneous curvature and the type of emulsion resulting from the process. In this case, the affinity of Tween 80 (non-ionic surfactant) for the oil phase increases as the temperature rises, leading to the preferential formation of an A/O (oil-in-water) emulsion. Conversely, at lower temperatures, the process reverses, with Tween 80 exhibiting a greater affinity for water, thus forming an O/A (water-in-oil) emulsion. This phenomenon occurs because the solubility of Tween 80 is influenced by hydrogen bonds, which weaken at higher temperatures [31,50,51].

Due to the difficulty of dispersing plant derivatives such as fatty amides in aqueous media, their incorporation into nanoformulations can help overcome this challenge [32]. Silk fibroin (SF), an amphiphilic protein derived from *Bombyx mori*, has shown great potential in nanocarrier systems due to its ability to stabilize water-in-oil formulations. Its hydrophobic domains interact with fatty acid amides, while the hydrophilic segments associate with the aqueous phase, enhancing dispersion and reducing interfacial tension. This contributes to improved physicochemical stability and bioavailability of lipophilic compounds. In our study, the inclusion of SF in the NLC formulations resulted in enhanced stability parameters and more uniform particle size distribution, as well as improved selective cytotoxicity against 4T1 breast cancer cells. These findings align with previous reports highlighting SF’s biocompatibility, low immunogenicity, and structural regulatory properties [38], further supporting its role as a functional component in drug delivery systems [52,53,54]. To achieve better integration of silk fibroin with fatty amides, they were homogenized and heated together with myristic acid to a temperature 5–10 °C above their melting point [55]. This heating process aims to produce a fine dispersion of an oil-in-water emulsion [56].

Particle size variations may have been influenced by the length and type of fatty acid derivative chains used in the formulation [57]. Based on these values, it can be inferred that NLC-FAA/SF exhibited a less dispersed particle size distribution, making the nanoformulations less susceptible to delamination or precipitation, and providing greater stability to the NLC [58].

The stabilization of protein-based nanoformulations is governed by the properties of the layers adsorbed onto the surface of the oil droplets [59]. In this regard, when adsorbed, protein molecules may unfold and reorient their amino groups, with hydrophobic groups aligning with the oil molecules and hydrophilic groups aligning with the aqueous phase, forming a viscoelastic or gelatinous layer at the oil/water interface [60].

It is worth noting that systems with an average size ranging from 50 to 200 nm are preferred for potential intravenous administration, as biodistribution is strongly influenced by particle size [61]. Particles smaller than 10 nm can be rapidly eliminated through glomerular filtration, while particles larger than 300 nm may be recognized by the mononuclear phagocytic system, leading to opsonization and removal from circulation [62]. In this context, particle sizes exceeding 200 nm can significantly affect circulation time, as this is influenced by the capillary size of the circulatory system [63].

The ZP evaluates the electrostatic repulsion between equally charged particles. It is important to clarify that while ZP values above +30 mV or below −30 mV are generally considered indicative of high colloidal stability due to strong electrostatic repulsion, nanoparticle stability is not governed solely by ZP. In our formulation, the NLC-FAA/SF system exhibited ZP values ranging from −8.33 ± 7.64 mV to −8.3 ± 12.0 mV, which may appear low under the criteria for electrostatic stabilization. However, these values must be interpreted considering the nature of the stabilizing agents used. The formulation includes Tween 80, a non-ionic surfactant, and silk fibroin, an amphiphilic protein that forms a viscoelastic steric barrier at the oil–water interface. This steric stabilization mechanism is effective even at lower ZP values, as it prevents particle aggregation through spatial hindrance rather than electrostatic repulsion. The stability of the formulation is further supported by the absence of significant changes in particle size and polydispersity index over 30 days of storage (Figure 4). Therefore, the observed physical stability results from a combined steric and mild electrostatic stabilization, as supported by previous literature, and should not be assessed based solely on ZP thresholds [64]. Additionally, long organic chains, such as those of the FAA present in the formulation, can create a steric barrier that prevents interaction between the nanoparticles [65].

The results also demonstrate the expected behavior of the nanoparticle in controlling release and enabling the encapsulation of poorly soluble substances. This is supported by the sustained release profile, with more than 40% of the drug retained within the nanoparticle after 24 h.

NLCs are biocompatible and/or biodegradable lipid-based nanoparticles widely employed in cancer therapeutics. Composed of solid and liquid lipids with a surfactant solution forming the core matrix, NLCs are ideal for encapsulating hydrophobic drugs and co-encapsulating various compounds. Their lipid core effectively delivers oils or biological agents. For instance, NLCs encapsulating 1′-acetoxychavicol acetate, a hydrophobic ester, have shown enhanced cellular uptake, modulated release, and efficacy against PC-3 prostate cancer cells. Similarly, Carvalho et al. (2022) [66] developed an NLC platform using copaiba oil for docetaxel co-encapsulation in breast cancer treatment. This platform demonstrated stability for over 360 days with >99% encapsulation efficiency and activity against 4T1 and MCF-7 breast cancer cell lines [67,68,69].

Silk fibroin is a well-established biodegradable, biocompatible, and non-inflammatory material with high blood compatibility. Studies confirm that silk fibroin nanoparticles exhibit low inflammatory responses and minimal coagulant properties. Over 100 biomedical and research patents related to silk fibroin exist in the USA.

The incorporation of silk fibroin further enhanced the biocompatibility of the formulation and contributed to a sustained release profile, which may improve bioavailability while minimizing systemic toxicity compared to lipid-only carriers [38].

In comparison to polymeric nanoparticles—renowned for their precision in particle size and drug release modulation—NLCs-FAA/SF benefit from their lipid-based architecture, which closely resembles cellular membranes. This structural mimicry facilitates cellular uptake and promotes efficient intracellular delivery of the active compounds [5]. The synergistic combination of cytotoxic FAAs with silk fibroin, functioning as a structural and release-modulating biopolymer, presents a compelling strategy for targeted and sustained cancer therapy by integrating the therapeutic benefits of both components [54].

This behavior is likely due to the presence of compounds in the medium that can interact with the fatty acids in the nanosystem, as previously reported [70,71].

The evaluation of colloidal stability in biological media did not show a statistically significant difference when compared to the physicochemical evaluation. However, the slight increase in colloidal size observed in both NLC formulations incubated in murine plasma and FBS can be attributed to the characteristics of these complex biological matrices. Specifically, the interaction of NLC with components such as serum proteins, globulins, biomolecules, ionic salts, and amino acids may have influenced the hydrodynamic behavior of the NLC, leading to slight instability due to the adsorption of these matrix components. This interaction can result in the formation of aggregates, potentially affecting the in vitro behavior [62]. It is worth noting that these results remain within the acceptable limits set by the American Pharmacopoeia for potential intravenous administration of injectable lipid formulations, which require a size of less than 500 nm or 0.5 μm [72].

Endocannabinoids are widely associated with cytotoxic activity, including in breast cancer [73,74]. One study reported that anandamide had an IC50 value of 1.5 ± 0.3 μM after 48 h of treatment in EFM-19 breast carcinoma cells, and the IC50 for MCF-7 cells was 0.5 μM. The study also reported the cytotoxic activity of both endocannabinoids and synthetic cannabinoids in the EFM-19 cell line. In a similar study, Melck et al. (2000) [75] reported an IC50 value of 1.4 ± 0.9 μM for the MCF-7 cell line and 1.9 ± 0.2 μM for the T-47D breast cancer cell line using anandamide, with an IC50 of 1.4 ± 0.3 μM for the 2-arachidonoylglycerol derivative in the MCF-7 cell line. We can infer that the cytotoxic effect of NLC-FAA/SF is due to the isobutylamides in andiroba oil, which are cannabinomimetics that interact with the CB2 receptor. CB2 expression is known to be increased in breast cancer, regardless of the subtype [76].

Breast cancer is one of the most prevalent cancers, and the search for treatments is of utmost importance [77,78]. As reported here, the cytotoxic activity in the 4T1 breast cancer cell line is significant, as it is a triple-negative model, one of the types with the highest morbidity and mortality [79,80]. Additionally, this model can be associated with other drugs; for instance, Slivicki et al. (2019) [81] demonstrated synergism and reduced neuropathic pain in the 4T1 breast cancer model by combining a fatty acid amide hydrolase inhibitor, which increases intracellular endocannabinoids, with paclitaxel.

Cannabinoid receptors CB1 and CB2 are G-protein-coupled receptors with distinct tissue distribution—CB1 predominantly in the central nervous system and CB2 primarily in immune-related tissues. Their activation modulates several intracellular signaling pathways and has therapeutic relevance in oncology [82,83,84].

Our docking findings suggest that unsaturated FAAs interact more strongly with both CB1 and CB2 than their saturated counterparts, likely due to enhanced molecular flexibility and conformational adaptability, which favor better accommodation in the receptor binding sites. Specifically, residues such as Phe200, Trp356, Phe268, and Val196—identified as critical for CB1 activation [85]—were involved in the interactions with unsaturated FAAs.

Similar observations were made for CB2, with key residues such as Phe87, Phe183, Trp194, and Ser285 being consistently engaged, corroborating previous studies by Pawar et al. (2022) and Omar et al. (2022) [41,42]. Additionally, comparison with docking results from analogs such as LS-61176 and cannabidiol [43] further validates the relevance of the observed interactions in our study.

The unfavorable interactions observed in saturated FAA docking may be attributed to steric hindrance and suboptimal spatial fitting within the binding cavity, reducing binding affinity. These structural findings align with previous literature suggesting that lipid unsaturation enhances membrane fluidity and receptor interaction, which can be advantageous for therapeutic modulation.

Despite promising affinity profiles, the poor aqueous solubility of FAAs presents a limitation for clinical translation. The use of nanostructured lipid carriers (NLCs) offers a viable solution by improving solubility, prolonging systemic circulation, and enabling co-delivery strategies. Encapsulation with silk fibroin further improves biocompatibility and provides controlled release properties [44,45,46].

Taken together, our results highlight that unsaturated FAAs show strong potential as CB1 and CB2 ligands, particularly when formulated within lipid-based nanocarriers. This dual strategy—molecular targeting combined with nanotechnological delivery—represents a promising approach for the development of cannabinoid-based therapies in breast cancer and potentially other malignancies.

## 4. Materials and Methods

### 4.1. Chemicals and Reagents

Isobutylamine (99.5%), Amano lipase from *Pseudomonas fluorescens* (20,000 U·g^−1^, CAS No. 9001-62-1), myristic acid (95%), and hexane (98%) were purchased from Sigma-Aldrich (São Paulo, Brazil). Polysorbate 80 (Tween 80) was purchased from Praid Produtos Químicos Ltda (São Paulo, Brazil). Ethanol (99%) was acquired from Solven (São Paulo, Brazil), and deuterated chloroform (CDCl_3_) was provided by the Cambridge Isotope Laboratories. Andiroba oil was obtained from Amazon Oil the Rainforest Company (Ananindeua, Brazil). RPMI 1640 medium, DMEM, fetal bovine serum, penicillin, streptomycin, and trypsin-EDTA 0.25% were sourced from Gibco-Invitrogen (São Paulo, Brazil). All other chemicals used in this study were of analytical grade.

### 4.2. Direct Amidation Reaction to Obtain Fatty Amides

A direct amidation reaction was performed between andiroba oil (1.0 mL) and ethanolamine (3.0 mL), using 10% Amano lipase from *Pseudomonas fluorescens* (LPF) as the catalyst. The reaction was maintained under magnetic stirring (300 rpm, 40 ± 2 °C) for 24 h. At the end of the reaction, the enzyme was filtered out, and the mixture was washed with ethyl acetate (3 × 1.0 mL). The filtrate was evaporated under reduced pressure, and the fatty amide (FAA) was purified by silica gel column chromatography, using n-hexane:ethyl acetate (8:2) as the elution solvent. The reaction product was characterized by gas chromatography–mass spectrometry (GC-MS) and nuclear magnetic resonance (^1^H and ^13^C NMR).

### 4.3. Silk Fibroin (SF) Solution

A silk fibroin solution was prepared according to the method created by Maciel Ferreira et al. 2014 [86]. The silkworm cocoon (3.0 g, obtained from Bratac, Londrina, Brazil) was treated in a solution of Na_2_CO_3_ (2%, w/v), boiling for 30 min. The resulting fibers were filtered and washed with distilled water (3 × 500 mL). Subsequently, the silk fiber was dissolved in a ternary solution (50 mL) of H_2_O:EtOH:CaCl_2_ (8:2:1 molar ratio) at 30 °C for 4 h. This mixture was then dialyzed (using a cellulose membrane with an exclusion limit of 16 kDa, from Viskase, Atibaia, Brazil) for 3 days at room temperature, with the water being changed every 24 h. The fibroin solution was centrifuged (6000 rpm for 10 min) to remove impurities and larger particles. The concentration of the silk fibroin solution was adjusted to 2% (w/w).

### 4.4. Preparation of the Nanostructured Lipid Carrier Containing FAA and SF (NLC-FAA/SF)

The NLC-FAA/SF was prepared using the homogenization method with stirring and heating [56]. The formulation composition is shown in Table 4. The aqueous and oily phases were heated separately, both to 65 °C; the aqueous phase was heated in a water bath, while the oily phase was heated on a heating plate. The aqueous phase was then slowly added to the oily phase, and the formulation was stirred and heated for 45 min. Without further heating, the formulation was left under stirring for 24 h. Finally, it was subjected to centrifugation at 20,000rpm for 30 min. The formed pellet was washed with buffer solution.

### 4.5. Gas Chromatography with Mass Spectrometry Detection (GC-MS)

Chromatographic analyses of FAA from AO were performed using a gas chromatograph (GCMS-QP 2010, Sâo Paulo, Brazil) equipped with an AOC-20i automatic injection sampler (Shimadzu, Sâo Paulo, Brazil). Detection was carried out using electron impact ionization with a Shimadzu MS2010 Plus detector (Sâo Paulo, Brazil), operating at an ionization energy of 70 eV. Mass fragments were detected within a range of 50 to 550 Da. Separation was achieved using a fused silica capillary column (RTX-5MS model (Restek, Bellefonte, PA, USA), with an internal diameter of 0.25 mm, 30 m length, and 0.25 µm film thickness) with a helium flow of 1.03 mL.min^−1^. The sample was dissolved in dichloromethane at a concentration of 2 µg·mL^−1^ and analyzed under the following experimental conditions: injector temperature set at 210 °C, detector temperature at 250 °C, and helium as the carrier gas with a flow rate of 3.0 mL·min^−1^. Injection was performed in split mode with a split ratio of 1:15. The column temperature program was 90 °C, followed by a linear increase at a rate of 6 °C·min^−1^ until reaching 250 °C. The final temperature was maintained isothermally for 5 min, resulting in a total analysis time of approximately 33.67 min [87].

### 4.6. Fourier Transform Infrared Analysis (FTIR)

FTIR spectroscopy was employed for the structural characterization of the samples. Spectra were recorded using Shimadzu IRAffinity spectrometers (Sâo Paulo, Brazil)for both OA and NLC-FAA samples. Following drying, a thin layer of each sample was spread onto a watch glass and evaporated at 40 °C under a stream of air to yield a dry lipidic powder. The dried material was then finely ground and mixed with potassium bromide (KBr) at a ratio of 2 mg of sample to 200 mg of dry KBr. The mixture was homogenized by grinding for approximately 5 min and then compressed using a hydraulic press to form thin KBr pellets. Transmittance measurements were conducted in the spectral range of 4000 to 400 cm^−1^, with a resolution of 4 cm^−1^ [88].

### 4.7. Nuclear Magnetic Resonance (^13^C NMR)

The ^13^C NMR spectra of AO and FAA were obtained using an Agilent Technologies 500/54 Premium Shielded spectromete (Sâo Paulo, Brazil). Each sample was dissolved in 600 μL of chloroform (CDCl_3)_. Chemical shifts are reported in ppm relative to TMS as the internal standard or the residual solvent peaks of the deuterated solvent [88].

### 4.8. Particle Size, Polydispersity Index (PdI), Zeta Potential, and pH

Dynamic light scattering (DLS) analysis was conducted using a Zetasizer Nano ZS90 (Malvern, UK) to determine the particle size (DS), polydispersity index (PdI), and zeta potential (ZP). Samples were diluted 100-fold in ultrapure water, transferred directly into a glass cuvette, and analyzed. A linear temperature ramp was programmed as follows: starting at 25 °C and increasing in increments of 5 °C until reaching a final temperature of 70 °C. The hydrogen potential (pH) of the NLC was measured using a pH meter (Digimed DM 20, Ribeirão Preto, Brazil). All measurements were performed in triplicate, and the results are expressed as mean ± standard deviation [89].

### 4.9. Drug Release

The drug release study was conducted using the dialysis method. A cellulose membrane weight cut-off of 14Kda and a diameter of 21 mm (Sigma-Aldrich, St Louis, MO, USA) were employed for the experiment. The nanoparticles were placed inside the dialysis bag, and a 5% (v/v) aqueous alcohol solution was used as the receiving medium. Samples were collected at predetermined time intervals (15, 30, 60, 120, 240, and 1440 min) over 24 h, with the receptor medium being replaced after each sampling. The samples, prepared in triplicate, were analyzed by Fourier transform infrared (FTIR) spectroscopy, as described in Section 4.6. Thin films were prepared using potassium bromide (KBr) discs, and the main isobutylamide peaks were identified and analyzed [90].

Fatty acid amide release from nanoemulsions was quantified by ATR-FTIR spectroscopy (Nicolet iS50, Thermo Fisher, Dreieich, Germany), monitoring the integrated areas of CH_3_ (2955 cm^−1^), CH_2_ (2922 cm^−1^), Amide I (1653 cm^−1^), Amide II (1545 cm^−1^), and PO_2_ (1239 cm^−1^) bands (Ceylani T, Teker HT, Samgane G, Gurbanov R. Intermittent fasting-induced biomolecular modifications in rat tissues detected by ATR-FTIR spectroscopy and machine learning algorithms) [33]. Calibration curves (0.05–1.0 µg/mL) confirmed linearity, with validation showing precision, accuracy, specificity, and stability per ICH Q2(R1) guidelines [91]. The acyl chain length index (A2922/A2955) was calculated to assess fatty acid structural changes during release.

### 4.10. Colloidal Stability

To predict the in vivo behavior of the evaluated nanostructures, the dilution stability of the white nanostructured lipid carriers (NLCw) and NLC-FAA/SF was assessed in various biological fluids [92]. The carriers were diluted at a ratio of 1:4 in the following solutions: (NaCl, 0.9% w/v), phosphate buffer (pH 7.4), fetal bovine serum (FBS), Dulbecco’s modified Eagle medium (DMEM), and mouse plasma. The samples were incubated at 37 °C under constant agitation at 150 RPM for 24 h. Aliquots were collected at predetermined time intervals, and the average diameter of the nanostructures was measured using DLS [89].

### 4.11. Cell Culture

The 4T1 murine breast cancer cell line (ATCC^®^ CRL-2539™- São Paulo, Brazil) was cultured in complete RPMI 1640 medium, while non-tumor human fibroblasts (NTHFs) derived from primary gingival tissue culture were grown in DMEM, both supplemented with 10% fetal bovine serum, 100 IU/mL penicillin, and 100 μg/mL streptomycin. The cells were maintained in a humidified incubator at 37 °C with a 5% CO_2_ atmosphere, grown to confluence, and harvested by trypsinization. The non-tumor human fibroblasts (NTHFs) from primary gingival tissue culture were kindly provided by Prof. Cláudia Maria Oliveira Simões (Laboratory of Virology, Universidade Federal de Santa Catarina, Florianópolis, Brazil). The use of these cells was approved by the Research Ethics Committee of the Universidade Federal de Santa Catarina under protocol number 021/2009.

### 4.12. Cell Viability

The cytotoxicity study was conducted using the sulforhodamine B assay, where the dye binds proportionally to the proteins of viable cells. For the assay, 5000 cells per well were plated for the 4T1 cell line, and 10,000 cells per well were plated for the fibroblast lineage. The cells were allowed to adhere and grow for 24 h. After this period, the cells were treated with the following: lipid nanoparticles containing fatty acid amides and silk fibroin (NLC-FAA/SF), andiroba oil (AO), white nanostructured lipid carriers fibroin, or DMSO (solvent control) at varying concentrations (25–0.19 µg/mL). The treatments were diluted in the culture medium before application [93].

The treatments were incubated for 48 h at 37 °C with 5% CO_2_ atmosphere. After incubation, 10% trichloroacetic acid (TCA) was added to each well, and the plates were stored at 4 °C for 1 h. The plates were then washed with Milli-Q^®^ water and allowed to dry. Subsequently, the cells were stained with 0.4% (w/v) sulforhodamine B in 1% acetic acid for 30 min at 25 °C. After staining, the plates were washed with 1% acetic acid to remove unbound dye. The plates were dried again, and the bound dye was solubilized using 10 mM Tris-Base buffer. The optical density was measured using a SpectraMax Plus 384 spectrophotometer (Molecular Devices, Sunnyvale, CA, USA) at a wavelength of 510 nm. The experiments were performed in triplicate, with three replicates per plate, totaling nine replicates per condition. The concentration required to inhibit 50% of cell viability (IC_50_) was determined using a dose–response curve.

### 4.13. Obtaining Molecular Docking

For the molecular docking study, the Genetic Optimization for Ligand Docking (GOLD) 2020.1 program was used to analyze the possible interactions between the target and ligand. This program employs a genetic algorithm (GA), which optimizes docking times and determines the most favorable docking pose. However, before conducting the docking study itself, it is necessary to validate the target. This process involves a comparison between experimental and theoretical studies to verify whether the program can reproduce similar results. This is achieved by calculating the root mean square deviation (RMSD) value, where a value below 2Å is considered acceptable, as it indicates that the program was able to identify the ligand in the active site, like the experimental study. Additionally, the validation process also provides data regarding the centroids and the radius of the active site, which are then used in the docking study [94,95].

**Figure 13:**
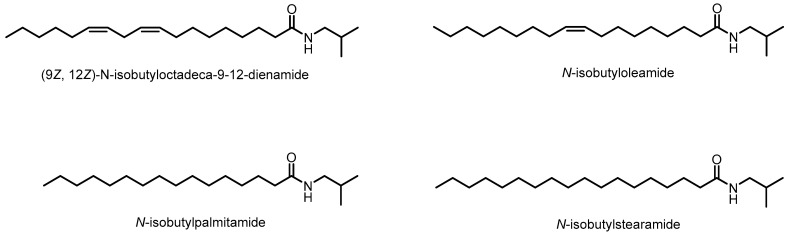
Molecular structures of the fatty acid amides.

The crystallographic structures of each target (CB1 and CB2) were obtained from the Protein Data Bank (PDB), with CB1 having the code 5XR8, complexed with the agonist (6~{a}~{R},9~{R},10~{a}~{R})-9-(hydroxymethyl)-3-(8-isothiocyanato-2-methyl-octan-2-yl)-6,6-dimethyl-6~{a},7,8,9,10,10~{a}-hexahydrobenzo[c]chromen-1-ol (Hua et al., 2017) [40], and CB2 having the code 8GUR, complexed with the ligand 2-[(1R,2R,5R)-5-hydroxy-2-(3-hydroxypropyl)cyclohexyl]-5-(2-methyloctan-2-yl)phenol [96]. Following target validation, the docking analysis between the fatty acid amides (Figure 13) and the CB1 and CB2 receptors was then conducted.

### 4.14. Statistical Analysis

Statistical analyses were performed using GraphPad PRISM software, version 8.00 (GraphPad Software Inc., La Jolla, CA, USA). Differences among experimental groups were analyzed using one-way analysis of variance (ANOVA) followed by Tukey’s post hoc test. All data met the assumptions of normal distribution and homogeneity of variance. *p*-values < 0.05 were considered statistically significant. The data are presented as mean ± standard deviation (SD).

## 5. Conclusions

This study successfully synthesized and characterized fatty acid amides (FAAs) from andiroba oil, confirming their structure via FTIR, ^13^C NMR, and GC-MS. The subsequent incorporation of these FAAs with silk fibroin (SF) into nanostructured lipid carriers (NLCs) resulted in formulations with excellent physicochemical and colloidal stability. Crucially, the NLC-FAA/SF demonstrated significant selective cytotoxic activity against 4T1 murine breast cancer cells (IC_50_ = 0.18 ± 0.06 μg/mL) while exhibiting minimal toxicity to healthy human fibroblasts. Molecular docking studies further elucidated the potential mechanism of action, revealing favorable interactions between the FAAs and cannabinoid receptors CB1 and CB2, with unsaturated FAAs showing superior binding and stability.

This work represents a significant advancement in breast cancer therapy by introducing a novel nanoplatform that combines the therapeutic potential of andiroba-derived FAAs with the biocompatibility and stability offered by silk fibroin. The selective cytotoxicity and promising molecular interactions underscore the originality and potential of this approach for targeted drug delivery.

Future research should focus on validating these findings through in vivo studies to confirm efficacy and safety in a living system. Further exploration of other molecular targets and optimization of the NLC-FAA/SF formulation are also warranted to enhance its clinical applicability and expand its therapeutic scope.

## Figures and Tables

**Table 1:** Mean sizes and PdI of NLCw and NLC-FAA/SF.

Nanostructured Lipid Carrier	Size (nm) T = 0	Size (nm) T = 1440	PdI T = 0	PdI T = 1440
NLC-FAA/SF	131.0 ± 10.15	130.90 ± 08.61	0.18 ± 0.01	0.21 ± 0.04
NLC-FAA/SF + NaCl	138.1 ± 9.51	139.20 ± 16.51	0.22 ± 0.03	0.21 ± 0.04
NLC-FAA/SF + PBS	131.6 ± 10.50	131.40 ± 09.77	0.19 ± 0.01	0.20 ± 0.04
NLC-FAA/SF + DMEM	146.4 ± 6.53	142.90 ± 08.86	0.22 ± 0.04	0.19 ± 0.03
NLC-FAA/SF + Plasma	164.6 ± 15.58	172.80 ± 26.71	0.22 ± 0.04	0.20 ± 0.03
NLC-FAA/SF + FBS	136.4 ± 7.03	141.30 ± 24.42	0.22 ± 0.08	0.20 ± 0.04
NLCw	160.7 ± 14.47	176.70 ± 18.07	0.21 ± 0.05	0.20 ± 0.04
NLCw + NaCl	182.6 ± 18.95	183.10 ± 12.50	0.23 ± 0.05	0.20 ± 0.03
NLCw + PBS	180.3 ± 18.11	181.00 ± 13.93	0.20 ± 0.04	0.20 ± 0.04
NLCw + DEMEM	182.7 ± 16.38	184.20 ± 11.05	0.19 ± 0.01	0.20 ± 0.03
NLCw + Plasma	175.9 ± 15.33	189.80 ± 22.60	0.19 ± 0.03	0.21 ± 0.04
NLCw + FBS	173.9 ± 10.02	187.20 ± 19.76	0.21 ± 0.06	0.21 ± 0.04

**Table 2:** Viability in 4T1 murine breast cancer cells (%).

Concentration (μg/mL) /Substances	NLC-FAA/SF	OA	NLCw	Fibroin
25.0	1.330 ± 0.262	2.135 ± 0.437	96.724 ± 1.559	109.255 ± 4.965
12.5	1.898 ± 0.247	1.973 ± 0.769	106.323 ± 0.281	108.237 ± 4.256
6.25	1.955 ± 0.753	2.631 ± 0.396	109.390 ± 8.625	106.702 ± 1.354
3.125	4.820 ± 1.827	8.440 ± 1.042	106.044 ± 3.366	105.547 ± 0.451
1.5625	8.644 ± 0.321	16.520 ± 3.957	103.183 ± 0.274	105.973 ± 2.386
0.7813	25.354 ± 2.797	33.934 ± 1.835	105.708 ± 2.689	111.170 ± 7.501
0.3906	37.168 ± 2.365	45.361 ± 2.941	106.116 ± 1.348	110.426 ± 8.683
0.1953	45.357 ± 0.751	59.349 ± 3.495	107.969 ± 0.509	115.897 ± 0.817

**Table 3:** Viability in healthy human fibroblasts (%).

Concentration (μg/mL) /Substances	NLC-FAA/SF	OA	NLCw	Fibroin
100.00	70.999 ± 5.551	85.790 ± 10.477	104.389 ± 3.945	109.244 ± 10.903
50.00	92.550 ± 5.853	98.849 ± 6.986	103.831 ± 2.879	107.345 ± 7.494
25.00	102.990 ± 2.412	105.487 ± 4.603	103.692 ± 2.321	111.721 ± 8.619
12.5	103.823 ± 1.183	103.299 ± 2.527	105.190 ± 2.814	117.083 ± 20.543
6.25	107.583 ± 4.026	106.886 ± 4.813	105.769 ± 1.269	112.055 ± 13.431
3.125	106.678 ± 5.107	106.411 ± 4.559	104.723 ± 1.240	107.951 ± 9.075
1.5625	103.173 ± 1.963	106.296 ± 4.720	104.487 ± 3.082	104.999 ± 3.090
0.7813	106.412 ± 5.644	105.472 ± 4.813	105.821 ± 4.968	105.911 ± 1.045

**Table 4:** Formulation of nanostructured systems.

NLC-FAA/SF			
Additive	Quantities (g)	Percentage (%)	Phase
Distilled water	4.835	96.5	Aqueous
SF solution (2%)	0.04	1	Aqueous
Tween 80	0.05	1	Aqueous
FAA	0.05	1	Oily
Myristic acid	0.025	0.5	Oily
Total q.s.p.	5	100	O/A
**NLCw**			
Additive	Quantities (g)	Percentage (%)	Phase
Distilled water	4.885	97.5	Aqueous
SF solution (2%)	0.04	1	Aqueous
Tween 80	0.05	1	Aqueous
Myristic acid	0.025	0,5	Oily
Total q.s.p.	5	100	O/A

## Data Availability

The original contributions presented in this study are included in the article/supplementary material. Further inquiries can be directed to the corresponding author.

## Supplementary Materials

The following supporting information can be downloaded at: https://www.mdpi.com/article/10.3390/molecules30163337/s1.
