# Supplementary material for: Evaluation of the Cytotoxic Activity of Nanostructured Lipid Carrier Systems for Fatty Acid Amides and Silk Fibroins in Breast Cancer Cell Lines
Source: Molecules. 2025 Aug 11;30(16):3337. doi: 10.3390/molecules30163337 (PMC12388404; doi:10.3390/molecules30163337)
Supplement: Supplementary file 1 [file molecules-30-03337-s001.zip › molecules-3717647-supplementary.pdf]

molecules

MDPI

Supplementary material

Evaluation of the Cytotoxic Activity of Nanostructured Lipid Carrier Systems for Fatty Acid Amides and Silk Fibroins in Breast Cancer Cell Lines

Sandro da Silva Borges [1], Sued Eustáquio Mendes Miranda [2], Victor Hugo de Souza Marinho [1], André Luís Branco de Barros [2], Sergio Yoshioka [3], Lorane Izabel da Silva Hage-Melim [4], Ana Carolina de Jesus Silva [4], Irlon Maciel Ferreira [1] and Anna Eliza Maciel de Faria Mota Oliveira [5,*]

[1] Biocatalysis and Applied Organic Synthesis Laboratory, Federal University of Amapá, University Campus Marco Zero do Equador, Rodovia Josmar Chaves Pinto, Km 02, Macapá 68902-280, AP, Brazil; ssilva.borges@hotmail.com (S.d.S.B.); virugo36@yahoo.com.br (V.H.d.S.M.); irlon.ferreira@unifap.br (I.M.F.)
[2] Department of Clinical and Toxicological Analyses, Federal University of Minas Gerais, Avenida Antonio Carlos, 6627, Belo Horizonte 31270-901, MG, Brazil; sued1989@ufmg.br (S.E.M.M.); albb@ufmg.br (A.L.B.d.B.)
[3] Biochemistry and Biomaterials Laboratory, Institute of Chemistry of São Carlos, University of São Paulo, Av. Trabalhador São-Carlense 400, São Carlos 13560-970, SP, Brazil; sergioy@iqsc.usp.br
[4] Laboratory of Pharmaceutical and Medicinal Chemistry, Federal University of Amapá, University Campus Marco Zero do Equador, Rodovia Josmar Chaves Pinto, Km 02, Macapá 68902-280, AP, Brazil; lorane@unifap.br (L.I.d.S.H.-M.); caroldejesus.farmacia@gmail.com (A.C.d.J.S.)
[5] Laboratory of Phytopharmaceutical Nanobiotechnology, Federal University of Amapá, University Campus Marco Zero do Equador, Rodovia Josmar Chaves Pinto, Km 02, Macapá 68902-280, AP, Brazil
* Correspondence: anna.oliveira@unifap.br

Table S1. Interactions and types of interactions between the ligands (9*Z*,12*Z*)-*N*-isobutyloctadeca-9,12-dienamide, *N*-isobutyloleamide, *N*-isobutylpalmitamide, and *N*-isobutylsteramide with the CB1 target.

Ligand	Amino Acid	Ligand Atom	Interaction	Type	Distance	Score
(9*Z*,12*Z*)-*N*-isobutyloctadeca-9,12-dienamide	Phe108	H56	Hydrogen Bond	Conventional Hydrogen Bond	1,68251	95,19
	Phe108	H57	Hydrogen Bond	Carbon Hydrogen Bond	2,93701	
	Trp279	H49	Hydrophobic	Pi-Sigma	2,62538	
	Val196	Ligand	Hydrophobic	Alkyl	4,66484	
	Val196	Ligand	Hydrophobic	Alkyl	4,69866	
	Leu193	C17	Hydrophobic	Alkyl	5,31572	
	Ile271	C17	Hydrophobic	Alkyl	4,75735	
	Leu276	C22	Hydrophobic	Alkyl	4,33815	
	Lys498	Ligand	Hydrophobic	Alkyl	3,90321	
	Phe170	Ligand	Hydrophobic	Pi-Alkyl	4,97042	
	Phe200	Ligand	Hydrophobic	Pi-Alkyl	4,70883	
	Phe268	Ligand	Hydrophobic	Pi-Alkyl	5,20958	
	Phe268	Ligand	Hydrophobic	Pi-Alkyl	4,50202	
	Trp279	Ligand	Hydrophobic	Pi-Alkyl	5,325	
	Trp279	C17	Hydrophobic	Pi-Alkyl	4,66995	
	Trp279	Ligand	Hydrophobic	Pi-Alkyl	4,44415	

 https://doi.org/10.3390/molecules30163337

	Trp279	C17	Hydrophobic	Pi-Alkyl	4,63908	
N-isobutyloleamide	Thr197	H58	Hydrogen Bond	Conventional Hydrogen Bond	2,02025	95,71
	Val196	Ligand	Hydrophobic	Alkyl	4,08037	
	Val196	Ligand	Hydrophobic	Alkyl	5,25313	
	Leu193	Ligand	Hydrophobic	Alkyl	5,13337	
	Lys498	C17	Hydrophobic	Alkyl	3,75236	
	Leu193	C22	Hydrophobic	Alkyl	5,13886	
	Ile271	C22	Hydrophobic	Alkyl	5,20815	
	Leu276	C22	Hydrophobic	Alkyl	5,25724	
	Phe170	Ligand	Hydrophobic	Pi-Alkyl	5,06027	
	Phe170	Ligand	Hydrophobic	Pi-Alkyl	5,39357	
	Phe177	Ligand	Hydrophobic	Pi-Alkyl	4,36504	
	Phe177	Ligand	Hydrophobic	Pi-Alkyl	4,90569	
	His178	Ligand	Hydrophobic	Pi-Alkyl	4,99619	
	Phe189	Ligand	Hydrophobic	Pi-Alkyl	5,00334	
	Phe268	C22	Hydrophobic	Pi-Alkyl	4,24104	
N-isobutylpalmitamideon	Ser173	H54	Hydrogen Bond	Conventional Hydrogen Bond	2,15827	71,94
	Val196	Ligand	Hydrophobic	Alkyl	4,74133	
	Val196	Ligand	Hydrophobic	Alkyl	4,91857	
	Val204	C15	Hydrophobic	Alkyl	3,51001	
	Val283	C15	Hydrophobic	Alkyl	3,62943	
	Lys192	C20	Hydrophobic	Alkyl	4,73998	
	Phe170	Ligand	Hydrophobic	Pi-Alkyl	4,77014	
	Phe200	Ligand	Hydrophobic	Pi-Alkyl	4,9036	
	Phe200	Ligand	Hydrophobic	Pi-Alkyl	5,15003	
	Trp279	Ligand	Hydrophobic	Pi-Alkyl	4,48622	
	Trp279	C15	Hydrophobic	Pi-Alkyl	5,1292	
	Trp478	Ligand	Hydrophobic	Pi-Alkyl	4,88804	
	Trp478	C15	Hydrophobic	Pi-Alkyl	4,00101	
	Val283	C16	Unfavorable	Unfavorable	2,29165	
	Val283	H53	Unfavorable	Unfavorable	1,51864	
	Val283	C16	Unfavorable	Unfavorable	1,73877	
	Val283	H53	Unfavorable	Unfavorable	0,831253	
N-isobutylsteramide	Phe108	H60	Hydrogen Bond	Conventional Hydrogen Bond	1,64747	71,71
	His178	H61	Hydrogen Bond	Carbon Hydrogen Bond	2,32896	
	Lys498	H62	Hydrogen Bond	Carbon Hydrogen Bond	3,00607	
	Phe200	H45	Hydrophobic	Pi-Sigma	2,99708	
	Val196	Ligand	Hydrophobic	Alkyl	4,75738	
	Val196	Ligand	Hydrophobic	Alkyl	4,34453	
	Ala502	C22	Hydrophobic	Alkyl	3,99725	
	Leu193	Ligand	Hydrophobic	Alkyl	4,96401	
	Val204	C17	Hydrophobic	Alkyl	4,22506	
	Val283	C17	Hydrophobic	Alkyl	3,58311	
	Val110	C22	Hydrophobic	Alkyl	4,10842	
	Lys498	C22	Hydrophobic	Alkyl	5,04008	
	Phe170	Ligand	Hydrophobic	Pi-Alkyl	5,33293	

	Phe177	Ligand	Hydrophobic	Pi-Alkyl	5,21871	
	His178	C22	Hydrophobic	Pi-Alkyl	4,98312	
	Phe200	Ligand	Hydrophobic	Pi-Alkyl	4,94308	
	Phe268	Ligand	Hydrophobic	Pi-Alkyl	5,46024	
	Phe268	Ligand	Hydrophobic	Pi-Alkyl	5,44649	
	Trp279	C17	Hydrophobic	Pi-Alkyl	5,23779	
	Trp279	Ligand	Hydrophobic	Pi-Alkyl	4,6707	
	Trp279	C17	Hydrophobic	Pi-Alkyl	4,4728	
	Trp478	Ligand	Hydrophobic	Pi-Alkyl	5,44839	
	Trp478	C17	Hydrophobic	Pi-Alkyl	4,52346	

Table S2. Interactions and types of interactions between the ligands (9*Z*,12*Z*)-N-isobutyloctadeca-9,12-dienamide, *N*-isobutyloleamide, *N*-isobutylpalmitamide, and **N-**isobutylsteramide with the CB2 target.

**Ligand**	**Ligand Atom**	**Ligand Atom**	**Interaction**	**Type**	**Distance**	**Score**
(9*Z*,12*Z*)-*N*-isobutyloctadeca-9,12-dienamide	Val113	Ligand	Hydrophobic	Alkyl	3,9993	91,02
	Val113	Ligand	Hydrophobic	Alkyl	4,80075	
	Ala282	C17	Hydrophobic	Alkyl	3,96121	
	Ile110	Ligand	Hydrophobic	Alkyl	4,42132	
	Ile186	C22	Hydrophobic	Alkyl	4,93302	
	Leu191	C22	Hydrophobic	Alkyl	4,63277	
	Met265	C22	Hydrophobic	Alkyl	5,4381	
	Phe87	Ligand	Hydrophobic	Pi-Alkyl	4,39318	
	Phe87	Ligand	Hydrophobic	Pi-Alkyl	4,15328	
	Phe91	Ligand	Hydrophobic	Pi-Alkyl	5,49687	
	Phe91	Ligand	Hydrophobic	Pi-Alkyl	4,15603	
	Phe91	Ligand	Hydrophobic	Pi-Alkyl	5,29479	
	Phe91	C17	Hydrophobic	Pi-Alkyl	5,14739	
	Phe94	Ligand	Hydrophobic	Pi-Alkyl	5,05589	
	His95	Ligand	Hydrophobic	Pi-Alkyl	4,37915	
	Phe183	C22	Hydrophobic	Pi-Alkyl	4,81591	
	Tyr190	C22	Hydrophobic	Pi-Alkyl	5,45177	
	Trp194	C22	Hydrophobic	Pi-Alkyl	5,31438	
	Trp194	C22	Hydrophobic	Pi-Alkyl	4,45786	
	Phe281	C17	Hydrophobic	Pi-Alkyl	4,8109	
N-isobutyloleamide	Tyr25	H58	Hydrogen Bond	Conventional Hydrogen Bond	1,74616	88,58
	Val113	Ligand	Hydrophobic	Alkyl	4,82109	
	Val113	Ligand	Hydrophobic	Alkyl	4,0613	
	Ala282	C22	Hydrophobic	Alkyl	4,38576	
	Ile110	Ligand	Hydrophobic	Alkyl	3,99507	
	Ile110	Ligand	Hydrophobic	Alkyl	4,89966	
	Ile186	C17	Hydrophobic	Alkyl	5,1466	
	Leu191	C17	Hydrophobic	Alkyl	4,80348	
	Met265	C17	Hydrophobic	Alkyl	5,2278	
	Leu182	C22	Hydrophobic	Alkyl	5,20981	

	Lys278	C22	Hydrophobic	Alkyl	4,6101	
	Phe87	Ligand	Hydrophobic	Pi-Alkyl	4,21771	
	Phe87	Ligand	Hydrophobic	Pi-Alkyl	4,93471	
	Phe91	Ligand	Hydrophobic	Pi-Alkyl	4,57947	
	Phe94	Ligand	Hydrophobic	Pi-Alkyl	5,03582	
	Phe183	Ligand	Hydrophobic	Pi-Alkyl	3,72837	
	Phe183	C17	Hydrophobic	Pi-Alkyl	4,55019	
	Trp194	C17	Hydrophobic	Pi-Alkyl	5,32646	
	Trp194	C17	Hydrophobic	Pi-Alkyl	4,31497	
N-isobutylpalmitamide	Tyr25	H54	Hydrogen Bond	Conventional Hydrogen Bond	1,763	53.34
	Trp258	H51	Hydrophobic	Pi-Sigma	4,36669	
	Val113	Ligand	Hydrophobic	Alkyl	5,36671	
	Ala282	C20	Hydrophobic	Alkyl	4,43801	
	Ile110	Ligand	Hydrophobic	Alkyl	4,96138	
	Ile110	Ligand	Hydrophobic	Alkyl	5,16079	
	Leu182	C20	Hydrophobic	Alkyl	5,20287	
	Lys278	C20	Hydrophobic	Alkyl	4,51039	
	Phe87	Ligand	Hydrophobic	Pi-Alkyl	5,27751	
	Phe117	Ligand	Hydrophobic	Pi-Alkyl	4,54633	
	Phe117	C15	Hydrophobic	Pi-Alkyl	4,54401	
	Phe183	Ligand	Hydrophobic	Pi-Alkyl	4,65499	
	Trp194	Ligand	Hydrophobic	Pi-Alkyl	5,19894	
	Trp194	C15	Hydrophobic	Pi-Alkyl	4,44784	
	Trp258	C15	Hydrophobic	Pi-Alkyl	4,55867	
	Phe117	H50	Unfavorable	Unfavorable	1,32906	
	Phe117	C15	Unfavorable	Unfavorable	1,67016	
	Phe117	H50	Unfavorable	Unfavorable	0,726279	
N-isobutylsteramide	Tyr25	H60	Hydrogen Bond	Conventional Hydrogen Bond	1,61239	51,28
	Pro184	Ligand	Hydrophobic	Alkyl	4,57766	
	Ala282	C22	Hydrophobic	Alkyl	4,47197	
	Ile110	Ligand	Hydrophobic	Alkyl	4,10126	
	Val121	C17	Hydrophobic	Alkyl	4,41378	
	Ile198	C17	Hydrophobic	Alkyl	4,49353	
	Leu182	C22	Hydrophobic	Alkyl	5,12756	
	Lys278	C22	Hydrophobic	Alkyl	4,52272	
	Phe117	Ligand	Hydrophobic	Pi-Alkyl	4,4992	
	Phe183	Ligand	Hydrophobic	Pi-Alkyl	4,74288	
	Phe183	Ligand	Hydrophobic	Pi-Alkyl	4,78673	
	Trp194	Ligand	Hydrophobic	Pi-Alkyl	4,17581	
	Trp194	C17	Hydrophobic	Pi-Alkyl	5,03204	
	Trp258	Ligand	Hydrophobic	Pi-Alkyl	4,9558	
	Trp258	C17	Hydrophobic	Pi-Alkyl	4,14644	
	Val121	C18	Unfavorable	Unfavorable	2,35236	
	Val121	H57	Unfavorable	Unfavorable	1,53894	
	Val121	C18	Unfavorable	Unfavorable	1,59301	
	Val121	H57	Unfavorable	Unfavorable	0,984264	
